# Retrograde trafficking of β-dystroglycan from the plasma membrane to the nucleus

**DOI:** 10.1038/s41598-017-09972-x

**Published:** 2017-08-29

**Authors:** Viridiana Gracida-Jiménez, Ricardo Mondragón-González, Griselda Vélez-Aguilera, Alejandra Vásquez-Limeta, Marco S. Laredo-Cisneros, Juan de Dios Gómez-López, Luis Vaca, Sarah C. Gourlay, Laura A. Jacobs, Steve J. Winder, Bulmaro Cisneros

**Affiliations:** 1 Departamento de Genética y Biología Molecular, Centro de Investigación y de Estudios Avanzados del Instituto Politécnico Nacional (CINVESTAV), Ciudad de México, Mexico, Mexico; 2 Laboratory of Protein Dynamics and Signaling, Center for Cancer Research-Frederick, National Cancer Institute, National Institutes of Health, Frederick, MD 21702 USA; 3 Instituto de Fisiología Celular, Universidad Nacional Autónoma de Mexico, Ciudad de Mexico, Mexico, Mexico; 4 Department of Biomedical Science, University of Sheffield, Western Bank, Sheffield, S10 2TN United Kingdom

## Abstract

β-Dystroglycan (β-DG) is a transmembrane protein with critical roles in cell adhesion, cytoskeleton remodeling and nuclear architecture. This functional diversity is attributed to the ability of β-DG to target to, and conform specific protein assemblies at the plasma membrane (PM) and nuclear envelope (NE). Although a classical NLS and importin α/β mediated nuclear import pathway has already been described for β-DG, the intracellular trafficking route by which β-DG reaches the nucleus is unknown. In this study, we demonstrated that β-DG undergoes retrograde intracellular trafficking from the PM to the nucleus via the endosome-ER network. Furthermore, we provided evidence indicating that the translocon complex Sec61 mediates the release of β-DG from the ER membrane, making it accessible for importins and nuclear import. Finally, we show that phosphorylation of β-DG at Tyr^890^ is a key stimulus for β-DG nuclear translocation. Collectively our data describe the retrograde intracellular trafficking route that β-DG follows from PM to the nucleus. This dual role for a cell adhesion receptor permits the cell to functionally connect the PM with the nucleus and represents to our knowledge the first example of a cell adhesion receptor exhibiting retrograde nuclear trafficking and having dual roles in PM and NE.

## Introduction

Dystroglycan (DG), a core component of the dystrophin-associated protein complex (DAPC), is an integral membrane receptor that links the extracellular matrix (ECM) with the actin-based cytoskeleton^1^. DG is encoded by the *DAG1* gene and translated as a polypeptide precursor that undergoes a post-translational proteolytic cleavage to generate two mature subunits (α- and β-DG)^2^. Although separated by this mechanism, α-DG and β-DG remain interacting with each other at the plasma membrane (PM) in a non-covalent fashion. α-DG is a peripheral membrane protein that interacts with ECM proteins through its highly glycosylated domains, while β-DG is a type 1 transmembrane protein that binds to the carboxy-terminal domain of α-DG on the extracellular side and to the actin cytoskeleton through its association with dystrophin and other cytolinker proteins^3, 4^. In addition to its primary role in the maintenance of the sarcolemmal stability, the DG complex has been shown to be involved in other cellular processes, including signal transduction and tissue morphogenesis^4–8^. Particularly β-DG, which modulates a plethora of cellular functions, working as a platform for cytoskeleton remodeling and cell adhesion systems, reviewed in ref. 4.

Unexpectedly, β-DG was found in the nucleus of diverse cell lines^9, 10^, which further extends its broad spectrum of functions. The nuclear import pathway of β-DG is dependent on the recognition of a nuclear localization signal (NLS), situated in the juxtamembrane region of β-DG, by the importin α2/β1 system^11, 12^; and this mechanism was further shown to be facilitated by ezrin-dependent cytoskeleton remodeling^11, 13^. Consistent with the notion of a novel role for β-DG in the nucleus, we demonstrated in our previous work that β-DG associates with different nuclear envelope (NE) proteins, including emerin and lamins A/C and B1, to critically regulate the nuclear structure and function in myoblasts^14^. In addition, the trafficking of β-DG to the nucleus has been implicated in the transcriptional regulation of androgen-responsive transcription factors in prostate cancer^15^. Taking all this evidence into account, β-DG must be regarded as a versatile protein playing physiological roles in both, plasma membrane (PM) and nucleus. Consequently, there must be a mechanism that assures the precise sorting of β-DG to distinct intracellular locations in response to cellular demands. However, such a mechanism remains to be elucidated.

In this study we characterize for the first time the nuclear trafficking pathway of β-DG in immortalized mouse C2C12 myoblasts. We demonstrate that β-DG undergoes retrograde trafficking from the PM to the nucleus, traveling through the endosome-endoplasmic reticulum (ER) network in a Sec61 translocon-dependent manner, prior to reaching the nucleus. In addition, we show that phosphorylation at Tyr^890^ favors the nuclear translocation of β-DG by enhancing its endosome-mediated internalization.

## Results

### β-DG nuclear targeting requires its previous transit from the endoplasmic reticulum (ER) to the Golgi apparatus

**Figure 1:**
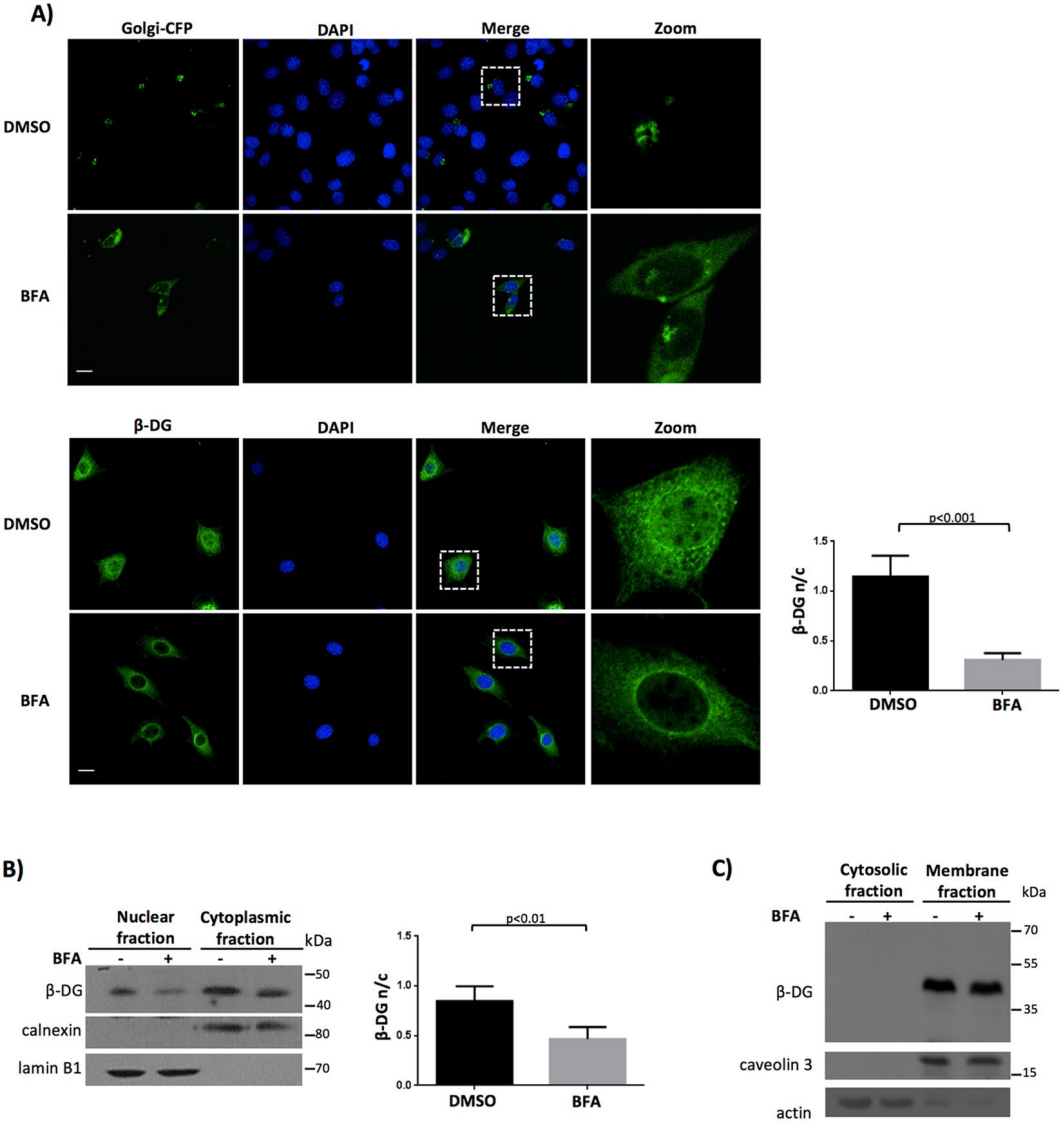
β-DG traffics from ER to Golgi prior to its nuclear localization. (**A**) C2C12 cells cultured on glass coverslips were transiently transfected to express the galactosyl-transferase-CFP fusion protein (Golgi-CFP) and 16 h post-transfection they were treated with BFA or vehicle alone (DMSO; see Methods). Afterwards, cells were fixed, stained with DAPI and further analyzed by CLSM (upper panel). C2C12 cells seeded on coverslips were treated with vehicle or BFA, fixed, immunolabeled for non-phosphorylated β-DG and counterstained with DAPI for nuclei visualization prior to analysis by CLSM (lower panel). Maximum intensity projections are shown. The fluorescence intensity in the nucleus and cytoplasm (F n/c ratio) was quantified to estimate β-DG nuclear accumulation (see Methods). Data shown in the graph (right) represent the mean +/− SD from three separate experiments (n = 30 cells), with p value denoting statistical significance (Student t-test). Scale bar 20 µm. (**B**) Control and BFA-treated cells were fractionated into cytoplasmic and nuclear extracts and further subjected to SDS-PAGE/Western blotting analysis using anti-β-DG antibodies for the immunodetetection of total β-DG. Membranes were striped and reprobed for calnexin (cytoplasmic marker) and lamin B1 (nuclear marker). Densitometric analysis of immunoblot autoradiograms was carried out and the relative levels of β-DG in the nucleus and cytoplasmic (n/c) were obtained. Results represent the mean +/− SD of 4 separate experiments, with *p* values indicating significant differences (Student t-test). (**C**) Vehicle- or BFA-treated cells were fractionated into cytosolic and total membrane extracts prior to being subjected to SDS-PAGE/Western blotting analysis, using anti-β-DG antibodies for total β-DG. Stripped membranes were reprobed for caveolin (membrane protein) and actin (cytosolic marker).

To decipher the molecular mechanisms underlying the nuclear trafficking of β-DG different strategies were approached. As DG is an extensively glycosylated protein which is normally synthesized in the endoplasmic reticulum (ER) and then transits the Golgi to acquire further modification, we first analyzed whether translocation of β-DG from the endoplasmic reticulum (ER) to the Golgi is a prerequisite for its subsequent nuclear localization. C2C12 cells were treated with brefeldin A (BFA), an inhibitor of the ER-Golgi anterograde transport, prior to examining β-DG subcellular distribution by confocal laser scanning microscopy (CLSM). Redistribution from Golgi to ER of the Golgi protein β-1,4-galactosyltransferasae fused to cyan fluorescent protein (Golgi-CFP) demonstrated the effectiveness of the BFA treatment (Fig. 1A, upper panels). Interestingly, BFA-treated cells also exhibited decreased nuclear staining of β-DG, compared with vehicle–treated cells; quantitative image analysis confirmed the decline of β-DG nuclear immunolabeling upon BFA treatment (F n/c of 1.14 and 0.31 for control and treated cells respectively; (Fig. 1A, lower panels). To support these findings with biochemical evidence, subcellular fractionation was carried out and nuclear and cytosolic fractions obtained from BFA- or vehicle-treated cells were subjected to immunoblotting analysis, using calnexin and lamin B1 as purity markers for cytoplasmic and nuclear fractions respectively. Consistent with the CLSM results, diminished nuclear levels of β-DG were observed in BFA-treated cells, with densitometry analysis showing ~40% decrease in the β-DG nuclear levels (n/c of 0.82 and 0.46 for control and treated cells respectively; Fig. 1B). By contrast, BFA treatment did not alter the levels of β-DG in the membrane fraction (Fig. 1C). Collectively these data indicate that the anterograde trafficking of β-DG through the Golgi is a necessary step for its subsequent translocation to the nucleus.

### β-DG undergoes endosomal-mediated retrograde trafficking from the PM to the nucleus

**Figure 2:**
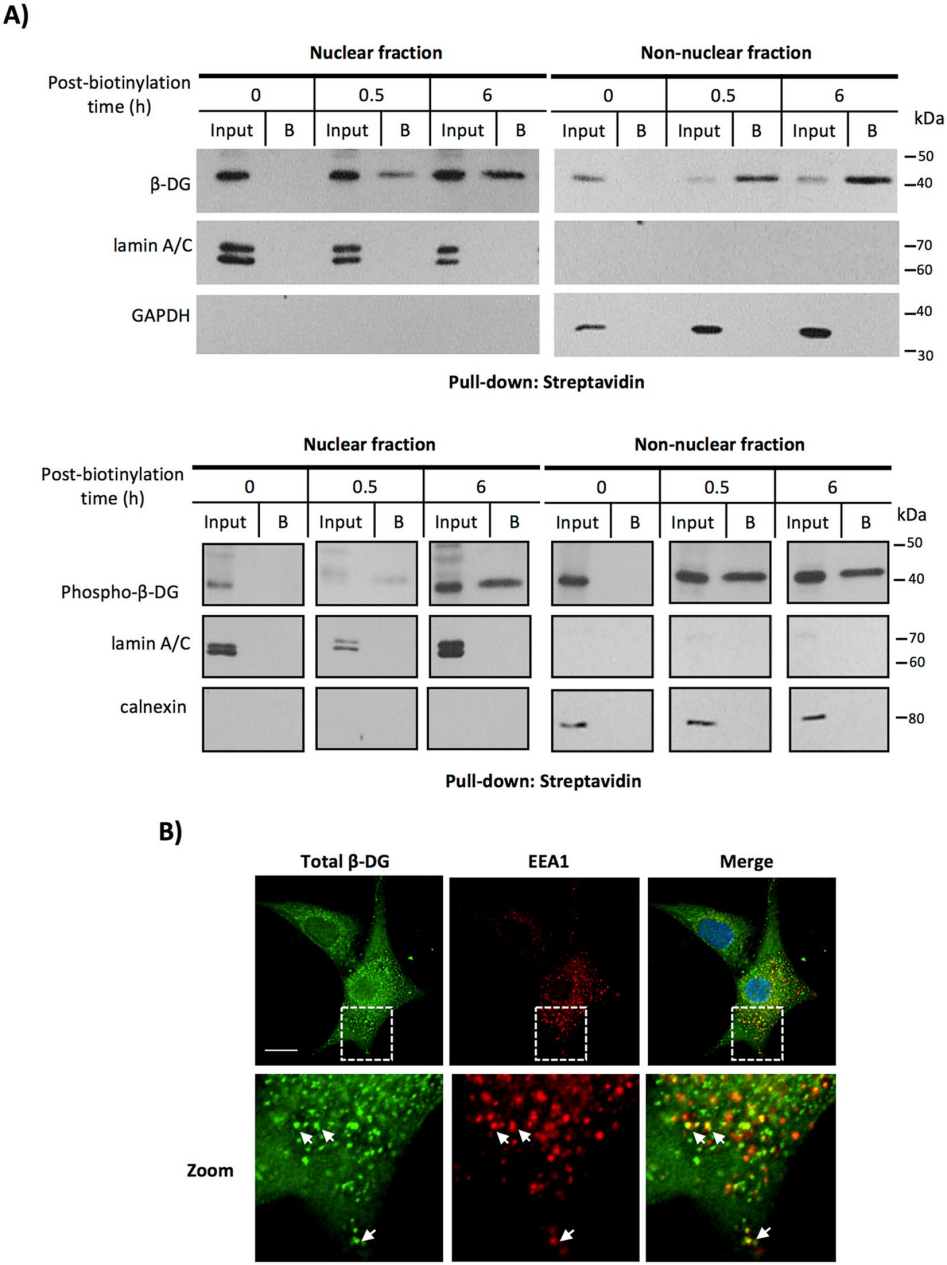
Nuclear β-DG derives from the PM. (**A**) C2C12 cells were incubated with biotin for the indicated time intervals to label cell surface proteins (see Methods). Cells were then subjected to subcellular fractionation to isolate nuclear and non-nuclear fractions and biotinylated proteins were pulled-down using streptavidin-agarose beads and analyzed by SDS-PAGE/Western blotting using primary antibodies for non-phosphorylated-β-DG (upper panel) and phosphorylated β-DG (lower panel). Lower panel blot was reorganized so that the time points order matched the ones shown in upper panel, original blot is shown in Supp. Figure 1. Input: immunoblotting analysis of cellular fractions prior to streptavidin-mediated pull-down. B: Bound/precipitated fraction. Membranes were stripped and reprobed for lamin A/C and GAPDH/calnexin as purity controls for nuclear and non-nuclear fractions respectively. (**B**) C2C12 cells cultured on glass coverslips were double-immunostained for total β-DG and the early endosomal marker EEA1. Nuclei were counterstained with DAPI prior to CLSM analysis. A typical single Z-section from three independent experiments is shown. Scale bar 20 µm.

β-DG is an integral membrane protein that localizes mainly at the PM. Therefore, we ascertained whether nuclear β-DG is derived from the PM upon internalization. To this end, C2C12 cell surface proteins were pulse-labeled with non-cell-permeable biotin for 30 minutes, followed by subcellular fractionation of treated cells into nuclear and non-nuclear fractions at 30 min and 6 h post-labeling. Biotinylated proteins were then precipitated with streptavidin agarose beads (Fig. 2A, upper panels). Purity of the cell fractions was validated by the absence of GAPDH in the nuclear fraction and lamin A/C in the non-nuclear fraction. Biotinylated β-DG was recovered in both nuclear and non-nuclear extracts at 30 min post-labeling and increased over 6 h, which implies that a fraction of nuclear β-DG is indeed derived from the PM. Neither lamin A/C nor GAPDH were present in the streptavidin-precipitated fractions, indicating that biotin-labeling was specific for cell surface proteins. It has been reported that phosphorylation of β-DG at Y890 (Y890 in mouse, Y892 in human: phospho-β-DG) triggers its detachment from the PM and internalization^16–18^. Thus, we tested whether phospho-β-DG derived from the PM could also be located in the nucleus. Consistent with this, we recovered phospho-β-DG in the non-nuclear fraction as expected, but also in the nuclear fraction of biotinylated proteins (Fig. 2A; lower panels).

**Figure 3:**
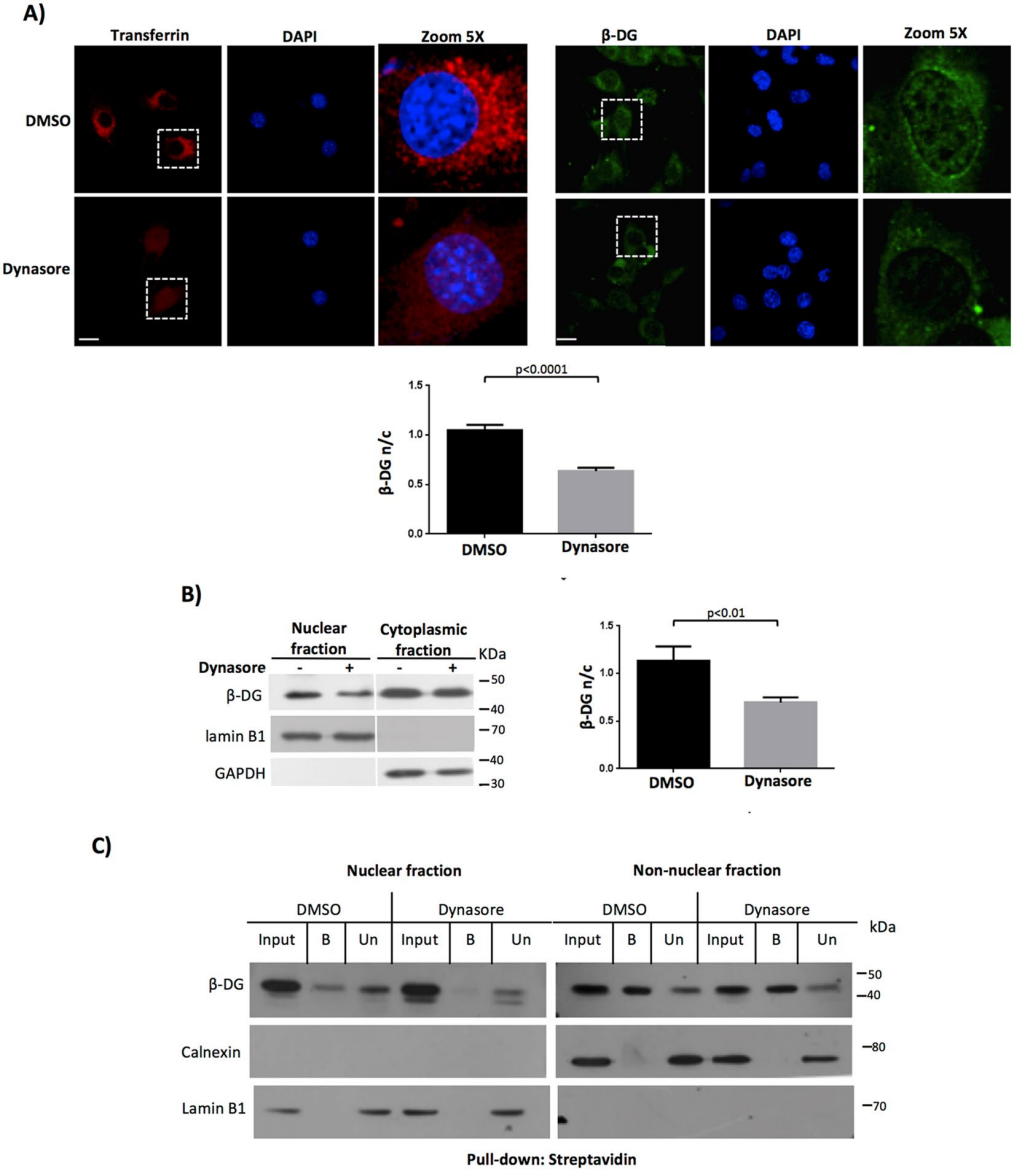
Inhibition of dynamin-dependent endocytosis reduces nuclear localization of β-DG. (**A**, left panel) Serum-starved C2C12 cells, seeded on coverslips, were treated with 40 µM dynasore (endocytosis inhibitor) or 0.05% DMSO (vehicle) for 30 min at 37 °C and then incubated for 5 min at 20 °C with Alexa594-transferrin (red). After 15 min at 37 °C, cells were fixed, stained with DAPI (nuclei) and transferrin uptake was monitored by CLSM analysis, with typical images shown (scale bar is 20 µm). (**A**, right panel) Cells treated with dynasore or DMSO, as above, were immunolabeled for β-DG and counterstained with DAPI prior to be imaged by CLSM, with typical Z-sections shown (scale bar is 20 µm). (A, lower panel). Nuclear accumulation of β-DG (F n/c) was estimated as described in Methods. Data correspond to mean +/− SD from a series of three separate experiments (n = 30 cells). (**B**) Cytoplasmic and nuclear extracts obtained from dynasore- or DMSO-treated cells were analyzed by Western blotting using anti-β-DG antibodies. Stripped membranes were reprobed for lamin A/C and GAPDH as purity and loading controls for nuclear and cytoplasmic extracts respectively. The nuclear/cytoplasmic ratio (n/c) of β-DG was estimated by densitometry analysis. Results represent the mean +/− SD for 3 separate experiments, with significant differences denoted by p values (Student t-test). (**C**) DMSO- and dynasore-treated cells were subjected to biotinylation assays as in Fig. 2. At 1 h post-biotinyation time, cells were fractionated into nuclear and non-nuclear fractions and pulled-down using streptavidin-agarose beads. Recovered and unbound proteins were analyzed by SDS-PAGE/Western blotting using primary antibodies for total β-DG. Membranes were stripped and reprobed for lamin A/C and calnexin; markers for nuclear and non-nuclear fractions respectively. Input: immunoblotting analysis of cellular fractions prior to pull-down. B, bound/precipitated fraction. Un, unbound fraction.

Internalization of β-DG has been described to occur through clathrin-mediated endocytosis and localize to transferrin-containing recycling endosomes^16^. Consistent with this, we revealed that β-DG partially co-localized with the early endosome antigen 1 (EEA1; Fig. 2B). Thus, we rationalized that blockage of the endosomal sorting by using dynasore, a specific inhibitor of the dynamin-dependent endocytosis pathway, might prevent nuclear translocation of β-DG. Dynamin is crucial for the scission of clathrin-coated pits from the cell surface^19^. To monitor clathrin-mediated endocytosis, C2C12 cells were subjected to a fluorescent transferrin-uptake assay. The uptake of transferrin was abrogated in dynasore-treated cells (Fig. 3A, left panels), which confirmed the effective inhibition of endocytosis. Consistent with the idea of endocytosis as the initial trafficking step for nuclear β-DG, dynasore treatment resulted in decreased nuclear labeling of β-DG with quantitative analysis showing F n/c of 1.0 and 0.6 for vehicle- and dynasore-treated cells respectively (Fig. 3A, right and lower panels). To confirm these results, subcellular fractionation was carried out in cells treated with dynasore or vehicle alone and cytoplasmic and nuclear fractions were further analyzed by WB. Lamin B1 (nuclear marker) and GAPDH (cytoplasmic marker) were immunodetected to validate the purity of the fractionation and they were also used as loading controls to normalize β-DG quantification. The nuclear level of β-DG decreased significantly upon dynasore treatment with densitometry analysis showing a 40% reduction in the n/c ratio (1.1 and 0.6 for DMSO- and dynasore-treated cells respectively; Fig. 3B). We did not observe a significant decrease of β-DG in the cytoplasmic fraction upon dynasore treatment most likely due to newly synthesized protein following its anterograde trafficking to the membrane and/or previously internalized β-DG in the cytoplasm. To demonstrate that dynasore treatment does indeed abrogate nuclear translocation of PM-derived β-DG, pulse-chase surface biotinylation was carried out followed by DMSO- and dynasore treatment of cells as above. Dynasore treatment resulted in a clear decrease in the nuclear levels of biotinylated β-DG at 30 min post-labeling, compared with control cells (Fig. 3C). Collectively these results show that β-DG and phospho-β-DG undergo an endocytosis-dependent retrograde trafficking from the PM to the nucleus.

### β-DG is translocated from the PM to the ER as part of its retrograde trafficking to the nucleus

**Figure 4:**
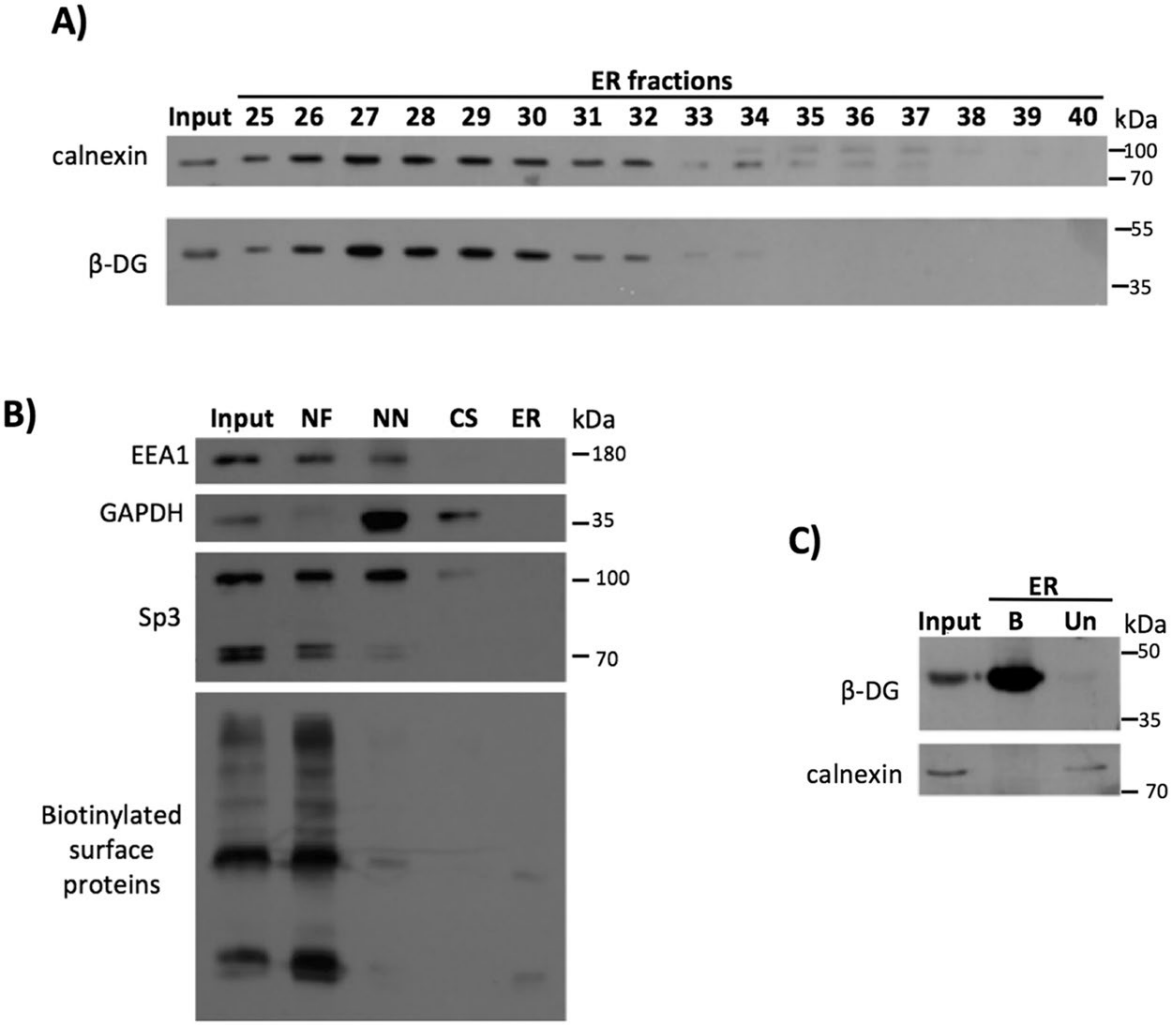
Retrograde trafficking of β-DG from the PM to the ER. (**A**) ER was purified using density gradient techniques (OptiPrep) and then ER fractions were immunoblotted for the ER marker calnexin or β-DG on the same membrane. (**B**) Verification of the purity of ER fractions: Aliquots from each step of the ER purification were analyzed by Western blotting using primary antibodies against EEA1 (early endosomal marker), GAPDH (cytosolic marker) and Sp3 (nuclear marker). As a PM marker, ER was isolated from biotinylated cells at 4 °C, the lysates were pulldown using streptavidin-agarose beads and then blotted with HRP-streptavidin. NF: Nuclear fraction; NN: Non-nuclear fraction; CS: Cytosolic fraction; ER: Endoplasmic reticulum fraction. (**C**) Cells were subjected to cell surface biotinylation and subsequently to ER purification using the OptiPrep gradient. The ER fractions were combined and biotinylated proteins were precipitated using streptavidin-agarose beads and then analyzed by SDS-PAGE/Western blotting with antibodies against β-DG and calnexin.

From the results shown above, we noticed that nuclear trafficking of β-DG resembles the retrograde transport of cell surface receptors that use an endosomal trafficking to the biosynthetic/secretory compartments, such as the endoplasmic reticulum (ER) and the Golgi apparatus, prior to entering to the nucleus^20–22^. Therefore, we set out to determine whether a retrograde trafficking step from PM to ER is part of the β-DG nuclear translocation pathway. To accomplish this, subcellular fractionation was carried out in order to obtain ER fractions through an OptiPrep density gradient purification, as described previously^20^. ER-enriched fractions were identified by the presence of calnexin, an ER-resident protein; interestingly, β-DG (43 kDa) was recovered in the same calnexin-containing fractions (Fig. 4A). We analyzed the presence of early endosome (EEA1), cytoplasmic (GAPDH), PM (biotinylated cell surface proteins) and nuclear (Sp3) proteins in each of the fractions that were recovered during the centrifugation steps of the protocol. We observed that the pellet collected from the first centrifugation step (NF) showed the presence of biotinylated proteins, mainly due to non-disrupted cells that were present in this fraction. Importantly, the ER fraction was free of all the proteins that were analyzed (Fig. 4B), which confirms its purification with none or minimal detectable contamination by other organelles. Since DG is synthesized in the ER as a single propeptide (90 kDa) that is proteolytically processed to generate α- and β-DG^23^, it was still necessary to ascertain whether a portion of β-DG found in the ER indeed corresponds to endocytosed and not to *de novo* synthesized protein. Cell surface proteins were labeled with biotin prior to ER purification and the collected ER fractions were combined and incubated with streptavidin-agarose beads to precipitate biotinylated proteins. Immunoblotting analysis revealed the presence of biotin-tagged β-DG in the ER (Fig. 4C), indicating that β-DG is transported from the PM to the ER as part of its retrograde trafficking to the nucleus. The absence of calnexin in the biotin precipitated fraction demonstrated that biotinylation was specific to cell surface proteins.

### The Sec61 translocon mediates the nuclear translocation of β-DG

**Figure 5:**
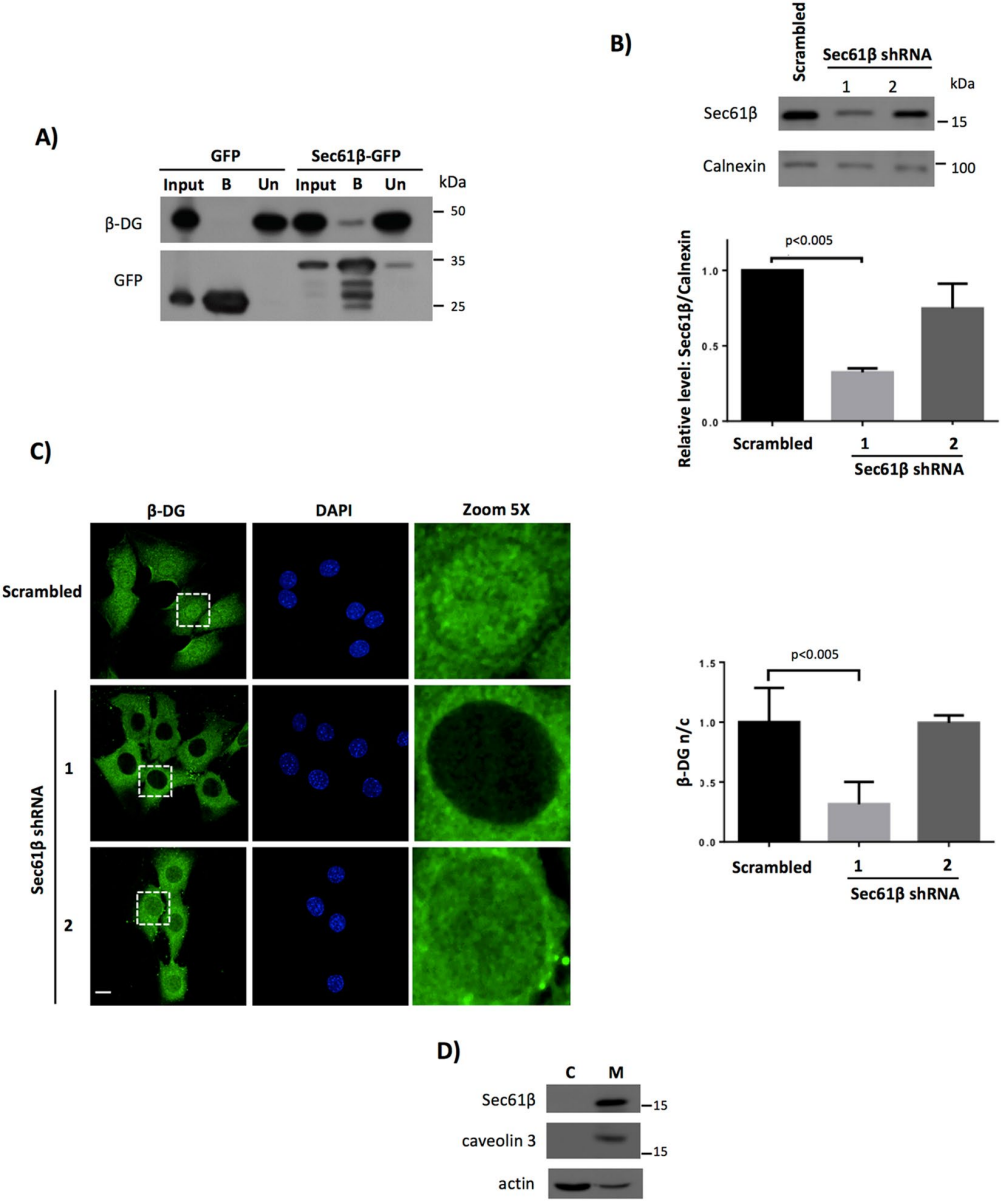
Nuclear translocation of β-DG is dependent on the Sec61 translocon. (**A**) C2C12 cells were transiently transfected to express Sec61β-GFP or GFP alone. The transfected cells were lysed 8 h post-transfection and immunoprecipitated using the GFP-Trap system; the precipitated proteins were analyzed by Western blot using antibodies against β-DG. Input corresponds to 5% of protein extract prior to immunoprecipitation; Un, unbound proteins; B, bound proteins. (**B**) Lysates from C2C12 cells stably transfected with vectors encoding shRNAs directed against mouse Sec61β mRNA (Sec61β shRNA1 and 2) or a scrambled shRNA (control) were analyzed by western blotting using antibodies against Sec61β and calnexin (loading control). (**C**) C2C12 cells expressing the scrambled shRNA or the Sec61β shRNAs (1 or 2) were cultured on glass coverslips, fixed, immunostained for total β-DG and counterstained with DAPI for nuclei visualization, prior to being analyzed by CLSM, with typical single Z-sections shown (scale bar is 20 µm). Quantitative analysis of the levels of β-DG nuclear accumulation (Fn/c ratio) was performed (right panel) and results represent the mean +/− SD for three separate experiments (n ≥ 50), with significant differences denoted by the p values (Student’s t-test). (**D**) Cells were fractionated to obtain cytosolic and membrane extracts. Distribution of Sec61β was analyzed by SDS-PAGE/Western blotting analysis using anti-Sec61β antibodies. Purity of cell fractions was analyzed by using primary antibodies against caveolin (membrane marker) and actin (cytosolic marker). C, cytosolic fraction; M, membrane fraction.

It has been shown that the retrograde trafficking of the epidermal growth factor receptor (EGFR) family proteins involves their interaction with the Sec61 translocon complex to escape from the ER/Golgi membranous environment and translocate to the nucleus^20, 24^ and reviewed in refs 22 and 25. We thus considered, whether the Sec61 translocon may also be involved in the retrograde nuclear trafficking of β-DG. As a first step, we analyzed whether β-DG could associate with the Sec61β translocon subunit by immunoprecipitation assays using the GFP-Trap approach. C2C12 cells were transfected to express Sec61β fused to GFP or GFP alone. We found that GFP-Sec61β but not GFP co-immunoprecipitates with the endogenous β-DG (Fig. 5A). Next, we evaluated the impact of Sec61β knockdown on β-DG nuclear localization. To this end, C2C12 cells were stably transfected to express small hairpin RNAs targeting mouse Sec61β mRNA (Sec61β shRNA 1–2) or a scrambled shRNA as control. Western blot analysis showed that only shRNA 1 was effective in lowering Sec61β expression (Fig. 5B); Depletion of Sec61β in the shRNA 1 cell culture resulted in ~75% decrease in β-DG nuclear immunostaining, whilst neither the scrambled nor the shRNA 2 affected β-DG nuclear localization (Fig. 5C), as shown by Fn/c quantification (right panel). Overall these data are consistent with the idea that trafficking of β-DG from the ER to the nucleus is dependent on the translocon Sec61. Furthermore, since all Sec61β was found associated with the membrane fraction of C2C12 cells (Fig. 5D), we speculate that Sec61 facilitates the release of β-DG from ER to be further recognized in its soluble form by importins.

### Phosphorylation at Tyr^890^ favors β-DG nuclear accumulation

**Figure 6:**
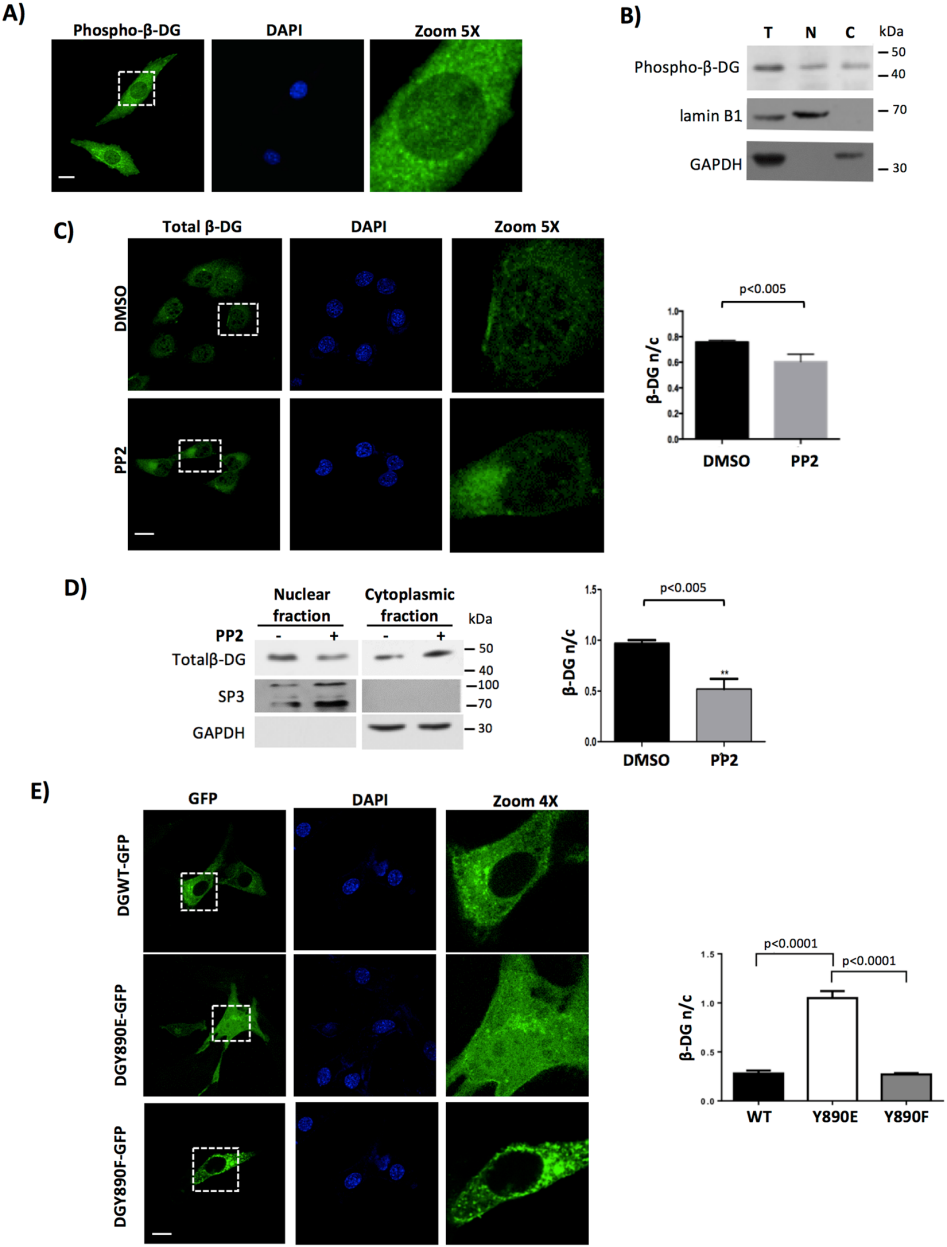
Src-dependent Tyrosine^890^ phosphorylation of β-DG drives its nuclear targeting. (**A**) C2C12 cells cultured on glass coverslips were treated with sodium orthovanadate (OV) for 3 h, fixed and immunostained using a phospho-specific antibody that recognizes Tyr^890^ phosphorylated β-DG. Nuclei were stained with DAPI (blue color) prior to CLMS analysis, with single optical Z-sections. Scale bar = 20 μm. (**B**) Cultures from OV-treated cells were fractionated into total (T), cytoplasmic (**C**) and nuclear (N) extracts and these extracts were separated by SDS-PAGE and subjected to Western blot analysis using phospho-β-DG antibodies. Nuclear (lamin B1) and cytoplasmic (GAPDH) protein markers were analyzed in parallel to validate the purity of the fractions. (**C**) C2C12 cells grown on glass coverslips were treated with PP2 or vehicle, fixed and immunolabeled for total β-DG and counterstaining with DAPI to visualize nuclei. Cells were imaged by CLMS and typical single Z-sections are shown (scale bar is 20 µm). Right panel. Quantitative analysis of CLMS images was carried out to obtain the nuclear to cytoplasmic fluorescence intensity of total β-DG (F n/c, see Methods). Results represent mean +/− SD for 3 separate experiments, with significant differences denoted by the p value (Student t-test). (**D**) Nuclear and cytoplasmic fractions obtained from control and PP2-treated cells were analyzed by SDS-PAGE/Western blotting using specific antibodies against total β-DG. Sp3 and GAPDH were used as loading controls for nuclear and cytoplasmic fractions respectively. Right panel. Nuclear to cytoplasmic ratio (n/c) of total β-DG was measured by densitometry analysis and data correspond to mean +/− SD for 3 independent experiments, with significant differences indicated by the p value (Student t-test). (**E**) C2C12 cells grown on glass coverslips were transiently transfected to express DGWT-GFP, DGY890E-GFP or DGY890F-GFP proteins. At 24 h post-transfection, cells were fixed, stained with DAPI and subjected to CLMS, with typical single optical Z-sections shown. Scale bar is 20 µM. Right panel. The F n/c ratio for DGWT-GFP, DGY890E-GFP and DGY890F-GFP was obtained and plotted. Results correspond to mean +/− SD for 3 separate experiments (n = 50 cells), with significant differences indicated by the p value (Student t-test).

As phosphorylation of β-DG at Tyr^890^ is thought to trigger its internalization from the plasma membrane to intracellular vesicular location (see above), we were prompted to examine whether this phosphorylation event may influence β-DG nuclear accumulation. As a first step, distribution of phosphorylated β-DG (phospho-β-DG) was assessed in cells treated with the phosphatase inhibitor sodium orthovanadate (OV), using an antibody that specifically recognizes the phosphorylated Tyr^890^ in β-DG. We observed that phospho-β-DG distributes throughout the cell, including plasma membrane, cytoplasm and nucleus (Fig. 6A), similar to what has been observed in prostate epithelium and prostate cell lines^15^. Subcellular fractionation followed by Western blot analysis confirmed the presence of phospho-β-DG in both cytoplasmic and nuclear extracts obtained from OV-treated cells (Fig. 6B). Immunodetection of nuclear (lamin B1) and cytosolic (GAPDH) markers validated the purity of the analyzed fractions. We next hypothesized that if phosphorylation plays a role in β-DG nuclear localization, it is expected that inhibition of c-Src, the kinase that phosphorylates β-DG at Tyr^890^ ^16^ would prevent β-DG nuclear accumulation. To approach this, C2C12 cells were treated with PP2 (specific c-Src inhibitor), PP3 (inactive analogue of PP2) or vehicle alone (DMSO), prior to analyzing β-DG distribution using either anti-phosho-β-DG antibody or the anti-β-DG antibody JAF, an antibody that recognizes β-DG irrespective of its phosphorylation status at Y890 (total β-DG) according to peptide recognition analysis using peptide SPOT arrays mapping the cytoplasmic domain of β-DG^26^ (Supp. Fig. 1A). The levels of phospho-β-DG were significantly decreased by PP2 but not by PP3 or DMSO, with no evident changes in the levels of total β-DG, which demonstrated the effectiveness of the treatment (Supplementary Figure 1B). Consistent with our hypothesis, decreased nuclear immunostaining of total β-DG was observed by CLSM analysis in PP2-treated cells, with quantitative image analysis (F n/c) confirming these observations (Fig. 6C). The WB analysis of cytosolic and nuclear extracts confirmed the decrease in the nuclear levels of total β-DG upon PP2 treatment, as evidenced by densitometry analysis showing n/c ratios of 0.9 and 0.5 for control and treated cells respectively (Fig. 6D).

To evaluate directly the effect of β-DG phosphorylation on its nuclear localization, the subcellular distribution of full-length (α/β-DG) GFP-tagged dystroglycan proteins, DGWT-GFP and its mutated variants that either mimic (DGY890E-GFP) or block (DGY890F-GFP) the Tyr^890^ phosphorylation, was analyzed at 24 h post-transfection by CLSM. Both DGWT-GFP and DGY890F-GFP were virtually absent from the nucleus, while DGY890E-GFP exhibited a robust nuclear accumulation; quantitative analysis (F n/c) corroborated these results (Fig. 6E). Collectively these data strongly suggest that Tyr^890^ phosphorylation positively modulates β-DG nuclear localization.

## Discussion

**Figure 7:**
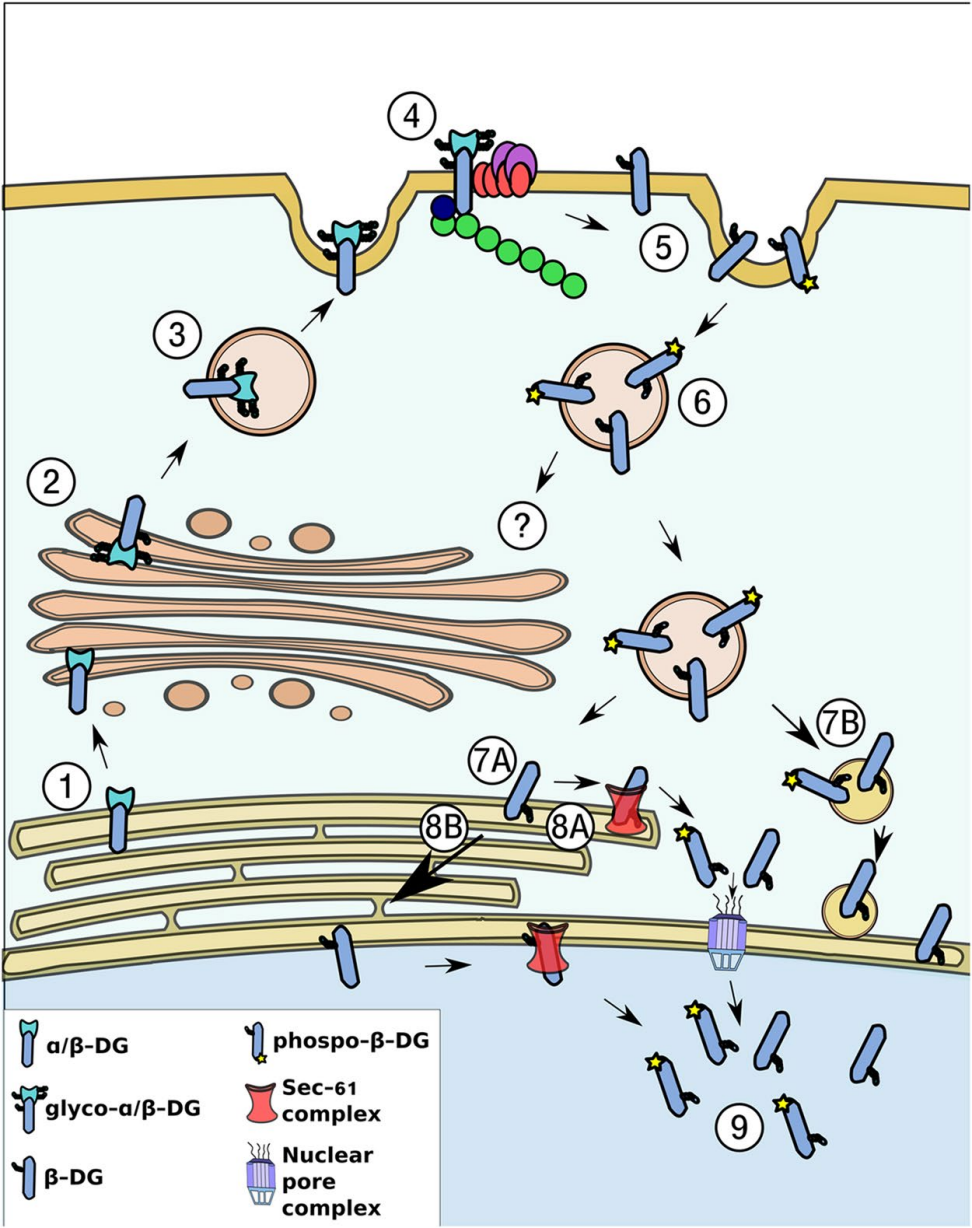
Schematic diagram of the retrograde trafficking of β-DG from the cell surface to the nucleus. (1) DG is synthesized in the ER as a precursor that undergoes a proteolytic cleavage to generate two subunits: α- and β-DG. (2) α-DG and β-DG maintain a non-covalent interaction and are transported from the ER to the Golgi apparatus, where both proteins are glycosylated (3) and then transported to the PM, where they interact with the DAPC (4). (5) β-DG is endocytosed from the PM, a process positively modulated by its phosphorylation on Tyr^890^. (7) β-DG is further translocated from the PM to the ER, and based on the β-DG-Sec61β interaction, it is likely that the Sec61 translocon releases β-DG from the ER membrane, prior to be recognized by the importin system to enter the nucleus through the NPC (8A-9). As both β-DG and Sec61 have been found in the NE, an alternative possibility is that β-DG moves from the ER to the NE by lateral diffusion to interact there with Sec61 to be directly delivered to the nucleoplasm (8B-9). Another alternative route for nuclear translocation is that β-DG-containing endosomes fuse directly to the nuclear membrane to discharge their contents in the nuclear envelope so that β-DG gets further translocated into the nucleoplasm (7B).

In this study we delineate for the first time a nuclear trafficking pathway for β-DG. We provide evidence showing that β-DG undergoes a retrograde transport from the cell surface to the nucleus, trafficking through endosomes and ER and using the Sec61 translocon to be translocated from the ER to the nucleus (Fig. 7). The following experimental evidence is consistent with this intracellular trafficking route. (a) Treatment with Brefeldin A, an inhibitor of the ER-Golgi anterograde transport pathway, decreased the nuclear accumulation of β-DG, which indicates that targeting of β-DG to the PM is a primary step for its subsequent nuclear translocation. (b) Inhibition of endocytosis by dynasore, which interferes with dynamin-dependent endocytosis by reversible blocking the GTPase activity of dynamin, prevented the nuclear translocation of PM-derived β-DG. (c) Cell surface biotinylated-β-DG was recovered in purified fractions of ER and nucleus, implying that a fraction of the β-DG found in these organelles is derived from the PM. Interestingly, a fraction of biotinylated β-DG was found in the nucleus 30 minutes after surface proteins biotin-labeling. This result is in agreement with the time that has been reported for PM receptors (e.g. EGFR) to reach the nucleus upon stimulation^27–29^ (d) The β-DG-Sec61β interaction was shown by immunoprecipitation assays whilst knockdown of Sec61β expression resulted in decreased β-DG nuclear localization, implying that trafficking of β-DG from the ER to the nucleus is dependent on the Sec61 translocon.

We previously established that nuclear import of β-DG is dependent on the recognition of a NLS, localized at its juxtamembrane domain (within amino acids 776–782) by the importin α2/β1 system^11^; in addition, we have shown that β-DG nuclear uptake is blocked by the *Agaricus bisporus* lectin ABL, which indicates that β-DG enters the nucleus through the nuclear pore complex (NPC). Therefore, in the light of the present results showing that β-DG uses the endosomal-ER network to reach the nucleus, we considered a mechanism that could enable β-DG to escape from the lipid bilayer to be further engaged in its soluble form by importins. In this regard, a previously unrecognized role for the Sec61 translocon complex has been described in the nuclear trafficking of the epidermal growth factor receptor (EGFR) family proteins, including EGFR and ErbB-2^20, 22, 27, 30^ reviewed in ref. 25. The Sec61 translocon complex, which comprises three transmembrane proteins, α, β and γ subunits, is classically associated with the ER, where it mediates the translocation of newly synthesized transmembrane proteins into the lipid bilayer. Also, Sec61 has been described to mediate the retrotranslocation of misfolded proteins from the lipid bilayer to the cytosol for proteosomal degradation^31^. It is thought that Sec61 facilitates the nuclear translocation of these cell surface receptors by releasing them from the ER membrane environment so that they can be later translocated into the nucleus as soluble proteins^20, 25^. Alternatively, due to the localization of Sec61 in the inner nuclear membrane (INM), it has been proposed that Sec61 may mediate nuclear trafficking of membrane-embedded proteins from the INM to the NPC^22^. The latter mechanism assumes that membrane-anchored proteins reach the INM prior to entering the nucleoplasm, which may occur by lateral diffusion from the ER to the INM or by direct delivery of endosomes to the INM^32^. Interestingly, we demonstrated that β-DG interacts with the Sec61β translocon subunit and that depletion of Sec61β levels restricts β-DG nuclear translocation, which strongly suggests that β-DG could be extracted from the lipid bilayer by the Sec61 translocon so that the protein becomes accessible to importins, which in turn mediate its translocation through the NPC to complete the nuclear trafficking process. As we found all Sec61β in the membrane fraction of C2C12 cells and since our previous study demonstrated that β-DG also localizes at the nuclear envelope, β-DG could be translocated by the Sec61 translocon from the ER or the INM, or both (Fig. 7). However, the lack of any obvious accumulation of β-DG in the INM when Sec61 is depleted, would tend to suggest that β-DG is translocated from the ER. The possibility that β-DG-containing endosomes fused directly to the nuclear envelope, which has also been shown to be a Sec61-dependent process^32^, cannot be ruled out.

The phosphorylation of β-DG on Tyr^892^ ^16, 17^ prevents its interaction with utrophin or dystrophin^33, 34^, and is a stimulus for its endocytic uptake and subsequent localization to early endosomes. Thus, it would be predicted that the nuclear localization of β-DG is positively modulated by this post-translational modification. Consistent with this idea, we showed that inhibition of c-Src, the kinase that phosphorylates β-DG^16^, resulted in decreased nuclear levels of β-DG, which implies that inhibition of phosphorylation interrupts the endosome-mediated internalization of β-DG, preventing its retrograde trafficking en route to the nucleus. Furthermore, while a β-DG variant mutant that is unable to be phosphorylated on Tyr^890^ (Y890F) was found to localize outside the nucleus, a mutant that mimics its phosphorylated state (Y890E) exhibited prominent nuclear localization, as evidenced by immunofluorescence.

The broad distribution of β-DG enables it to play diverse functions in the cell. As a DAPC component, β-DG serves as a platform for the organization of adhesion structures, including podosomes and focal adhesion structures^26, 35^, as well as integrin-mediated signaling cascades systems, including the ERK/MAP kinase pathway^4, 7^. In parallel, nuclear β-DG associates with NE components, including emerin and lamins A/C and B1, to preserve nuclear structure^14^ and indirectly modulate gene expression^15^. Therefore, an efficient anterograde/retrograde trafficking pathway is required for β-DG to maintain its correct distribution in the PM as well as the nucleus, and thereby to fulfill its important roles in both organelles. It is worth mentioning that unbalance in the nuclear levels of β-DG (depletion or overexpression) resulted in altered NE organization and activity^13^ (Vélez-Aguilera *et al*., unpublished results), which highlights the importance of a retrograde route for efficient delivery of β-DG to the nucleus. Nuclear trafficking of β-DG might thus serve as a mechanism to connect events at the plasma membrane with the nucleus, and to orchestrate nuclear activity (reorganization of the NE structure and/or modulation of NE-mediated gene expression) in response to cellular requirements. Nevertheless, identification of other cellular stimuli governing nuclear trafficking of β-DG as well as the whole functional consequences within the nucleus warrants further investigation.

Several transmembrane surface receptors are known to translocate to the nucleus, either in whole or in part. Cleavage of the intracellular domain (ICD) of several adhesion receptors, such as notch and CD44 and the translocation of the ICD to the nucleus is an integral part of their signaling mechanism. Like β-DG, the ICD of notch and CD44 have roles in regulating transcriptional activity in the nucleus^36–38^. Tyrosine kinase receptors such as EGFR and FGFR, and GPCRs, like the bradykinin B2 receptor, are translocated to the nucleus intact^39–43^. The role of the FGFR in the nucleus remains unclear, whereas translocation of the EGFR and bradykinin B2 receptors to the nucleus is believed to have a role in transcriptional regulation^22, 44^. β-DG is also translocated to the nucleus intact, where it functions in the NE as a nuclear cytoskeletal anchor^14^, in a manner similar to its role in the plasma membrane^4^. Thus, our findings reveal for the first time that the role of β-DG acting as a membrane-cytoskeletal anchor in both the plasma membrane and the inner nuclear membrane is linked by a retrograde trafficking pathway.

## Materials and Methods

### Cell culture and drug treatments

C2C12 cells were cultured as previously described^14^. Where indicated, cells were subjected to the following treatments: 2.5 µg/mL Brefeldin A (BFA; Cell Signaling Technology) or DMSO (vehicle) for 8 h; 2 mM sodium orthovanadate (Sigma, St. Louis, MO, USA) for 3 h; 10 µM PP2 or PP3 (Sigma-Aldrich, Missouri, USA,) or DMSO (vehicle) for 3 h in 5% FBS-supplemented DMEM. For fluorescent transferrin-uptake assays, cells grown on coverslips at 50% confluency were incubated with 80 µM dynasore (Sigma-Aldrich, Missouri, USA) or DMSO (vehicle) for 30 min at 37 °C in 5% FBS-supplemented DMEM and then incubated with medium containing Alexa 594-labeled transferrin (Molecular Probes, Thermo Fisher Scientific, Inc) for 5 min at 20 °C. After three washes, cells were transferred back to 37 °C incubation in fresh medium (endocytosis proceeds). 15 min later, cells were cooled to 4 °C, followed by an acid wash to remove the remaining transferrin still bound to the cell surface. Nuclei were stained with DAPI prior to confocal laser scanning microscopy (CLSM) analysis. For knockdown experiments, C2C12 cells were stably transfected with psi-mH1 vector expressing each of four different small hairpin RNAs (shRNAs) specific for mouse Sec61β mRNA, with a scrambled shRNA as a control (GeneCopoeia Inc. Rockville, MD). Transfected cells were cultured for 8–14 days in the presence of 2 µg/ml puromycin (Invitrogen, Carlsbad, CA, USA), prior to use for experiments.

### Plasmid, transfection and GFP-based precipitation

The following expression vectors were used; pECFP-N1-Golgi (Clonotech Laboratories, Inc); pAcGFP-C1-Sec61β (Addgene, Cambridge, MA); vectors expressing Y890F and Y890E variants of β-DG were generated using a QuickChange Site-Directed Mutagenesis kit (Stratagene) according to the manufacturer’s instructions with a full length α-/β-dystroglycan-GFP construct^45^ as template. Forward and reverse primers for the Y890F mutation were CACCCCCTCCGTTTGTTCCCCCTGCC and GGCAGGGGGAACAAACGGAGGGGGTG, and for the Y890E mutation were CGATCACCCCCTCCGGAAGTTCCCCCTGCCCC and GGGGCAGGGGGAACTTCCGGAGGGGGTGATCG respectively. Cells were transfected with the appropriate vector using lipofectamine 2000 (Invitrogen, Carlsbad, California, USA) following the provider’s protocol, and further analyzed at 24 h post-transfection. Lysates from C2C12 cells expressing GFP-tagged proteins were precipitated using the GFP-Trap® system (Chromotek, Germany) following the manufacturer’s instructions.

### Antibodies

The following primary antibodies were used. β-DG antibodies: JAF1, a rabbit polyclonal antibody^46^; 7D11, a mouse monoclonal antibody (Santa Cruz Biotechnology, Santa Cruz, CA., USA), MANDAG2, a mouse monoclonal antibody (Pereboev *et al*., 2001) and pY892.21.1, a mouse monoclonal antibody specific for phospho-β-DG (Santa Cruz Biotechnology, Santa Cruz, CA., USA). Rabbit polyclonal antibodies against: calnexin, lamin A/C, GAPDH, Sp3, lamin B1 and EEA1, a goat polyclonal antibody against caveolin (Santa Cruz Biotechnology (Santa Cruz, CA., USA) and a mouse monoclonal antibody against Sec. 61β (Abcam, Cambridge, UK) were utilized. Anti-actin mouse monoclonal antibody was a gift from Dr. Manuel Hernández (CINVESTAV. Mexico City). Epitope mapping of JAF1^46^ on β-DG peptide SPOT arrays was carried out as described previously^26^ using JAF1 antibody at a dilution of 1:400.

### Subcellular fractionation and western blotting

Isolation of total, cytoplasmic and nuclear protein extracts was carried out as previously described^14^. Fractionation into cytosol and total membrane extracts was carried as previously described^47^ with minor modifications. In brief, cells (8 × 10^6^) were washed and scraped in ice-cold PBS and spun for 5 min at 200 g. Cells were suspended in 0.5 ml cell lysis buffer buffer [10 mM HEPES pH 7.9,10 mM NaCl, 1 mM KH_2_PO_4_, 5 mM NaHCO_3_, 5 mM EDTA pH 8.0, 1 mM CaCl_2_, 0.5 mM PMSF and 1X Complete protease inhibitor cocktail (Roche Applied Science)]. Homogenization was carried out by applying 50 strokes with a Dounce homogenizer. Thereafter, 50 µl of 2.5 M sucrose was added to restore isotonic conditions. Cell homogenates were centrifuged at 6,300 g for 5 min and postnuclear supernatants were then centrifuged at 107,000 g for 30 min. The supernatant (cytosol) was collected and the resulting pellet (total membrane) was suspended in 0.2 ml of 1X RIPA buffer. Protein lysates were subjected to electrophoresis on 10% SDS-polyacrylamide gels and transferred onto nitrocellulose membranes (Hybond-N+, Amersham Pharmacia, GE Healthcare, Buckinghamshire, UK). Membranes were further blocked in TBST [100 mM Tris-HCl pH 8.0, 150 mM NaCl, 0.5% (v/v) Tween-20], with low-fat dried milk and incubated overnight at 4 °C with the corresponding primary antibodies. The specific protein signal was developed using the appropriate secondary antibodies and Enhanced Chemiluminescence (ECL™) Western blotting detection system (Amersham Pharmacia, GE Healthcare), according to the manufacturer’s instructions.

### Immunofluorescence and confocal microscopy analysis

C2C12 cells grown on coverslips were fixed with 4% PFA in PBS, permeabilized with 0.3% Triton X-100 in PBS, blocked with 0.5% gelatin-1.5% BSA in PBS and incubated overnight at 4 °C with the appropriate primary antibodies by following standard procedures. Cells were washed with PBS and incubated for 1 h at room temperature with fluorescein-conjugated goat anti-rabbit IgG, fluorescein-conjugated goat anti-mouse IgG or TRITC-conjugated goat anti-mouse IgG (Zymed Laboratories, Inc. San Francisco, CA, USA) and counterstained with 0.2 μg/μl DAPI (Sigma-Aldrich) for 10 min at room temperature to label the cell nuclei. Cell preparations were mounted on microscope slides with VectaShield (Vector Laboratories Inc. Burlingame, CA, USA) and further analyzed by confocal laser scanning microscope (CLSM; TCP-SP5, Leica Microsystems, Heidelberg, Germany) using a Plan Neo Fluor 63x (NA = 1.4) oil-immersion objective. Analyses of digitized images were carried out using Image J 1.62 software to determine the nuclear-cytoplasmic ratio (F n/c) as previously described (Suárez-Sanchez R *et al*., 2014).

### Biotinylation of cell surface proteins

Cell surface proteins were biotinylated with 0.25 mM sulfo-NHS-LC-biotin (Thermo-Fisher Scientific, Rockford, IL) at room temperature for 30 min, followed by incubation with 100 mM glycine in PBS to quench the reaction, as previously described (Wang YN *et al*.^22^). To isolate biotinylated proteins, treated-cells were subjected to cell fractionation (see above) and the total, nuclear or cytoplasmic extracts (250 µg) were incubated overnight at 4 °C with 20 uL of Streptavidin-conjugated agarose beads (Thermo-Fisher Scientific, Rockford, IL). Beads were then collected by centrifugation at 10,000 rpm for 5 min at 4 °C, washed tree times with ice cold 0.1% Triton X-100 in PBS and the biotinylated proteins bound to the streptavidin beads were solubilized in 6X SDS-PAGE sample buffer and boiled for 10 min at 95 °C prior to western blotting.

### Endoplasmic reticulum purification

ER Purification was carried out as previously described (Liao, HJ and Carpenter, G. 2007) with minor modifications, by using the OptiPrep density gradient system (Sigma-Aldrich, St Louis, Missouri, USA). Briefly, cells cultured on fifteen 100 mm dishes were harvested, washed with ice-cold PBS, resuspended in 1 ml of homogenization buffer (10 mM Tris-HCl pH 7.5, 250 mM Sucrose, 25 mM NaF, 10 mM Na3MO4, 2 mM Na2VO4, 1x complete protease inhibitor cocktail, 1 mM PMSF) and sonicated (3.5 microns, 10 secs, 3 times). The suspension was subsequently centrifuged at 12,000 g for 20 min at 4 °C and the pellet was collected as the nuclear fraction (NF) for subsequent analysis. The supernatant, corresponding to the non-nuclear fraction (NN), was transferred to an ultracentrifuge tube and centrifuged at 100,000 g for 60 min at 4 °C. The supernatant was collected as the cytosolic fraction (CS) for subsequent analysis. The pellet (microsomal fraction) was resuspended in homogenization buffer and mixed with 60% Optiprep to a final concentration of 20% Optiprep. The microsomal fraction was then centrifuged overnight at 200,000 g at 4 °C, to make an Optiprep density gradient. One-drop fractions were collected and analyzed by Western Blot; fractions enriched with Calnexin were considered as endoplasmic reticulum fractions.

## Electronic supplementary material

Supplementary figures
