# Publisher Correction: Retrograde trafficking of β-dystroglycan from the plasma membrane to the nucleus

**DOI:** 10.1038/s41598-018-36661-0

**Published:** 2018-12-07

**Authors:** Viridiana Gracida-Jiménez, Ricardo Mondragón-González, Griselda Vélez-Aguilera, Alejandra Vásquez-Limeta, Marco S. Laredo-Cisneros, Juan de Dios Gómez-López, Luis Vaca, Sarah C. Gourlay, Laura A. Jacobs, Steve J. Winder, Bulmaro Cisneros

**Affiliations:** 1 Departamento de Genética y Biología Molecular, Centro de Investigación y de Estudios Avanzados del Instituto Politécnico Nacional (CINVESTAV), Ciudad de México Mexico, Mexico; 2 Laboratory of Protein Dynamics and Signaling, Center for Cancer Research-Frederick, National Cancer Institute, National Institutes of Health, Frederick, MD 21702 USA; 3 Instituto de Fisiología Celular, Universidad Nacional Autónoma de Mexico, Ciudad de Mexico Mexico, Mexico; 4 Department of Biomedical Science, University of Sheffield, Western Bank, Sheffield, S10 2TN United Kingdom

Correction to: *Scientific Reports* 10.1038/s41598-017-09972-x, published online 29 August 2017

The original PDF version of this Article contained a truncated Figure 6. This error has now been corrected in the PDF version of the Article; the HTML version was correct from the time of publication.

