# Supplementary material for: Retrograde trafficking of β-dystroglycan from the plasma membrane to the nucleus
Source: Sci Rep. 2017 Aug 29;7:9906. doi: 10.1038/s41598-017-09972-x (PMC5575308; doi:10.1038/s41598-017-09972-x)
Supplement: Supplementary file 1 — Supplementary figures [file 41598_2017_9972_MOESM1_ESM.pdf]

RETROGRADE TRAFFICKING OF β-DYSTROGLYCAN FROM THE PLASMA MEMBRANE TO THE NUCLEUS

Supplementary information

Authors:
Viridiana Gracida-Jiménez[1]†, Ricardo Mondragón-González[1]†, Griselda Vélez-Aguilera[1], Alejandra Vásquez-Limeta[1,2], Marco S. Laredo-Cisneros[1], Juan de Dios Gómez-López[1], Luis Vaca[3], Sarah C. Gourlay[4], Laura A. Jacobs[4], Steve J. Winder[4] and Bulmaro Cisneros[1*]

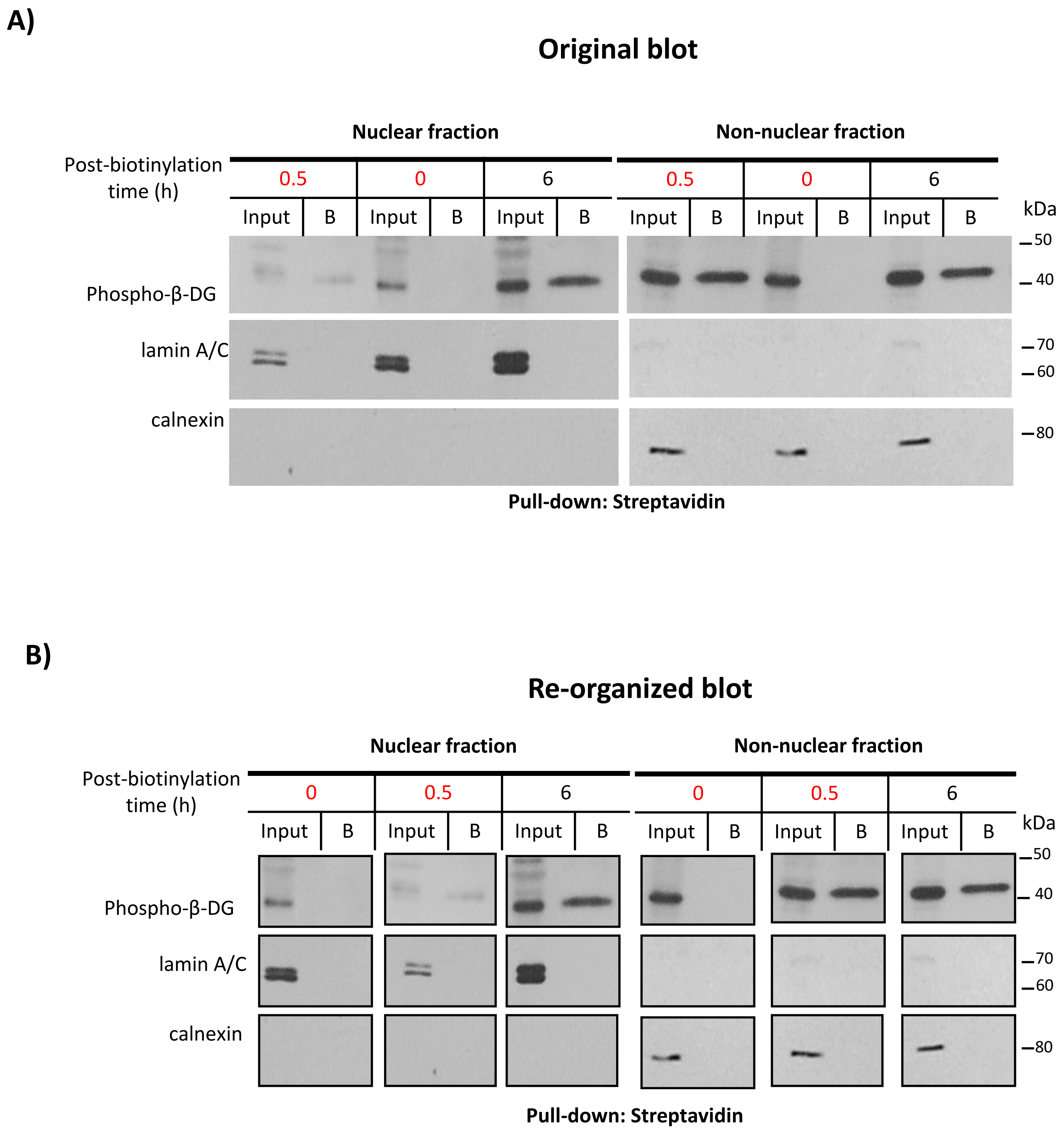

Supp. Fig. 1. Nuclear β-DG derives from the PM: Reorganization of lanes in the blot. In Fig 2. A (lower panel), lanes were reorganized from the original blot so that the post-biotinylation analysis time points matched with the ones shown in Fig. 2 A (upper panel). (A) In the original blot, samples were loaded in the following time points order: 0.5h, 0h, 6h. (B) Lanes were reorganized so that the time points were 0h, 0.5h and 6h.

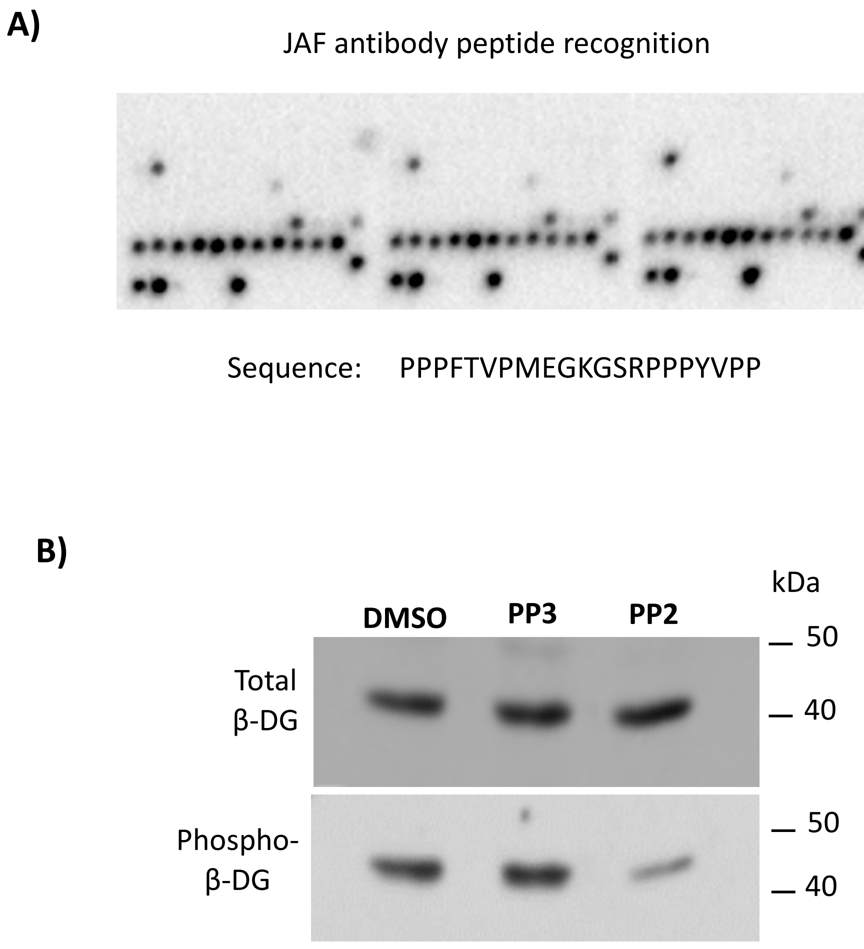

Supp. Fig. 2 Epitope-recognition mapping of JAF antibody and assessment of effectiveness of PP2 treatment on β-DG phosphorylation (A) We mapped the epitopes of β-DG recognized by JAF using peptide SPOT arrays to the entire cytoplasmic domain of β-DG and demonstrated that JAF bound to peptides in the extreme C-terminus of the cytoplasmic domain of β-DG. Although JAF1 was raised against the C-terminal 12 residues, it recognizes 21 residues. This is likely to be due to a repeated PPxxVP sequence, which may be the epitope recognized since it occurs twice in the 21 amino acids identified in the SPOT array. Thus, JAF is able to recognize individual peptides that did not include Y890 and so would not be affected by Y890 phosphorylation as is the case for Mandag2 for example, and can therefore faithfully report total β-DG levels. (B) In order to test the effectiveness of the c-Src inhibition treatment, we incubated C2C12 cells with PP2 (c-Src inhibitor) or its inactive analogue (PP3). Western blot analysis using an antibody specific to phospho-β-DG demonstrated that PP2 treatment conditions were indeed decreasing β-DG phosphorylation.
